# Bleeding with the Hollow Needle

**Published:** 1898-01

**Authors:** M. Casper


Bleeding with the Hollow Needle.—By M. Casper. In No. 13 of the *Berliner Thierarztliche Wochenschrift*, Professor Dieckerhoff describes a new process of bleeding in a manner which leaves the impression that this method is his discovery. For the protection of personal interests, and to state clearly the point at issue, I feel compelled to make the following reply: Already, since the year 1891, in procuring blood for the purpose of the serum at the Berlin Pathological Institute, I have conducted the blood out, in the case of larger and smaller animals, by means of a small hollow tube (canulus), which Hauptner made according to my instructions. The hollow needle used by Dieckerhoff differs from this only very slightly and very unessentially. My method was very soon adopted in Koch's Institute for Infectious Diseases.

At a meeting of the Kurhessiun Veterinarians I gave the following description of the same: I have the neck below the point of puncture compressed with a cord, then I cleanse the skin thoroughly with alcohol, and always keep the hollow needle or canulus in absolute alcohol. Then I stick the needle with a powerful thrust into the vein. The blood comes out in a bow-shaped stream, but cannot come in contact with the hand; from behind I draw out the needle while holding back the skin with two fingers of the left hand, and bleeding ceases almost immediately. In this manner I have let blood more than a thousand times without having had a single unfortunate result. I have also at times punctured the carotid, which can be easily recognized in the bright red color of the blood and through the pulsations in the stream itself.

In this case a large and hard swelling follows, but without injurious consequences, disappearing after a few days.

I can recommend this method with a good conscience to my colleagues. It can easily be performed, and looks more elegant and less cruel than bleeding with the lancet. In the case of cattle it is not so good, because the skin is so thick and the needle is hard to stick in.

In September, 1896, I showed this method to twenty colleagues, among them Prof. Dieckerhoff, who seemed much pleased. But in my innocence I did not suppose that he would afterward take occasion to describe the same as his discovery, without even mentioning my name.—*Deutsche Thierarzt. Wochenschrift*, April 10, 1897.

